# Determinants of comprehensive knowledge of mother-to-child transmission of HIV among currently married women in Somalia: analysis of the 2020 Somali Demographic and Health Survey

**DOI:** 10.1186/s12981-026-00928-8

**Published:** 2026-07-31

**Authors:** Liban Ali Mohamud, Abdirahman Hassan Hersi, Omar Abdisalam Yusuf, Abdikani Hussein Mohamed Kadie

**Affiliations:** 1 Research and Development Department, Salaam University, Mogadishu, Somalia; 2 Department of Statistics and Planning, SIMAD University, Mogadishu, Somalia

**Keywords:** Comprehensive knowledge, Mother-to-child transmission, HIV prevention, Reproductive-age women, Somalia

## Abstract

**Background:**

A significant proportion of HIV infections in infants results from vertical transmission, largely due to inadequate maternal knowledge of mother-to-child transmission (MTCT) and its prevention. Although several global studies have assessed women’s MTCT knowledge, nationally representative evidence from Somalia remains limited. This study addresses this gap by examining factors associated with comprehensive knowledge of MTCT of HIV among currently married women aged 15–49 years in Somalia.

**Methods:**

Multivariable logistic regression was used to examine the association between comprehensive MTCT knowledge of HIV and sociodemographic factors using data from the 2018–2019 SDHS.

**Results:**

Overall, 66.67% of currently married women had good knowledge of MTCT of HIV. Women residing in Cluster 7 (Gedo and Lower Juba) (AOR = 0.39, 95% CI 0.31–0.50) and Cluster 6 (Bay and Bakool) (AOR = 0.53, 95% CI 0.39–0.71), those who never listened to the radio (AOR = 0.71, 95% CI 0.57–0.88), and women in the richest wealth category (AOR = 0.72, 95% CI 0.59–0.87) were significantly less likely to have comprehensive MTCT knowledge.

**Conclusion:**

Policy interventions should focus on strengthening MTCT of HIV awareness through targeted education for younger women, those with limited media exposure, and women in Cluster 6 and Cluster 7. Expanding community-based and antenatal care-based HIV education is essential. Efforts should also reduce socioeconomic and regional disparities by improving access to HIV information among low-income and underserved populations to ensure equitable knowledge distribution.

## Introduction

HIV remains a significant global public health challenge, with an estimated 39 million people living with HIV (PLHIV) at the end of 2022 [1, 2]. A critical milestone in the HIV response is the elimination of vertical transmission (VT), also known as mother-to-child transmission (MTCT). Without intervention, the rate of VT can be as high as 15–45%, but this can be reduced to below 5% with effective antiretroviral therapy (ART) and comprehensive prevention of vertical transmission (PVT) programs [3, 4]. Despite global progress, VT continues to be the primary route of HIV infection in infants and young children, accounting for nearly 90% of new infections in this age group globally [4–6]. This burden is disproportionately borne by low-resource settings, particularly in sub-Saharan Africa (SSA), where health system fragilities, socioeconomic barriers, and gender inequalities limit the reach and effectiveness of PVT services [5].

Somalia exemplifies the challenges of combating HIV in a complex, resource-limited, and fragile state. The country experiences a low-level, concentrated epidemic, with a general adult HIV prevalence of 0.1% in 2019 [7, 8]. However, prevalence among key populations, such as women in high-risk settings, is significantly higher, reaching 4.5% in some urban centers [7]. The national HIV response is guided by the HIV National Strategic Plan (NSP) 2021–2023, which aligns with the global 90-90-90 targets. As of 2019, Somalia was not on track to meet these targets, with only 32% of PLHIV knowing their status, 79.4% of those diagnosed on ART, and 73.7% of those on ART achieving viral suppression [7, 9]. The PVT landscape in Somalia is characterized by a fragile health system and significant access barriers. Key health indicators reveal profound gaps; for instance, only 9.4% of births are attended by skilled health personnel, and the maternal mortality ratio is one of the highest in the world [8, 10, 11]. While the country has adopted the WHO-recommended “Option B+” strategy, ensuring lifelong ART for all HIV-positive pregnant women, the coverage and integration of these services within routine antenatal care (ANC) remain limited. The 2016 Service Availability and Readiness Assessment (SARA) found that only 15% of health facilities offering ANC provided HIV testing [7, 10].

A key pillar of preventing MTCT is comprehensive maternal knowledge. This includes accurate awareness that HIV can be transmitted during pregnancy, delivery, and breastfeeding, and the understanding that ART can dramatically reduce this risk [5, 12]. Studies across various settings have consistently linked inadequate knowledge of MTCT to factors such as low educational attainment, limited media exposure, and infrequent contact with health services [5, 12]. In Somalia, where cultural and religious norms mean that pregnancy predominantly occurs within marriage, currently married women represent the key demographic at risk of MTCT and are the primary target for PVT interventions. Their knowledge levels directly influence their ability to seek testing, adhere to ART, and adopt safer infant feeding practices [9, 13].

Although numerous studies [12, 14–17] conducted globally and in SSA countries have examined women’s knowledge of MTCT, particularly among pregnant women attending antenatal care and within broader female populations, there remains a lack of recent, nationally representative data investigating the determinants of comprehensive knowledge of VT among women. Therefore, this study aims to address this gap by analyzing the 2020 Somali Health and Demographic Survey (SHDS) [18] to determine the prevalence and identify the key socio-demographic and economic determinants of comprehensive knowledge of MTCT among currently married women. The findings are intended to provide critical evidence to inform and refine PMTCT strategies within the ongoing Somali National Strategic Plan (2021–2023), ultimately contributing to the elimination of mother-to-child transmission of HIV in the country. Furthermore, the study supports progress toward the sustainable development goals, particularly SDG 3 (Good Health and Well-being), which aims to end the AIDS epidemic and improve maternal and child health outcomes.

## Methods and data

### Study design and data source

This study uses a cross-sectional design based on secondary data from the 2018–2019 SDHS [18], conducted by the Ministry of Health of the Federal Government of Somalia in collaboration with the Somali National Bureau of Statistics. The survey is nationally representative and provides data on key demographic and health indicators, including fertility, infant and child mortality, reproductive behavior, maternal and child health, nutritional status, and HIV/AIDS awareness.

### Sampling framework

The sampling framework covered all eighteen pre-conflict regions of Somalia, which were stratified into urban, rural, and nomadic areas, except Banadir, which was entirely urban, resulting in 55 strata. Due to security constraints, all strata in Lower Shabelle and Middle Juba, and the rural and nomadic strata in Bay, were excluded, leaving 47 strata [8, 11, 17, 19–22]. A total of 10,525 enumeration areas (7488 urban and 3,037 rural) were identified using satellite imagery, of which 9136 were retained. For nomadic populations, a separate frame of 2521 temporary settlements was used. A three-stage stratified cluster sampling design was applied in urban and rural areas. Primary and secondary sampling units were selected using probability proportional to size, followed by systematic household sampling. Initially, 1433 primary sampling units were selected (770 urban, 488 rural, 175 nomadic). From these, 220 urban and 150 rural units were retained, along with all 175 nomadic units, yielding 545 clusters. In the final stage, 30 households per cluster were selected to produce reliable national estimates. Further methodological details are available in the SDHS 2018–2019 report [18, 22, 23].

### Study population

A total of 17,826 women were selected for the survey, of whom 16,486 eligible women were successfully interviewed, yielding a response rate of 92.5%. Among those interviewed, 11,876 were ever-married women and 4610 were never-married women. The present study focused on currently married women aged 15–49 years, resulting in an initial analytical sample of 10,324 women.

The primary outcome was comprehensive knowledge of mother-to-child transmission (MTCT) of HIV, operationalized as a composite measure based on three knowledge items: (1) awareness that HIV can be transmitted during pregnancy, (2) awareness that HIV can be transmitted during delivery, and (3) awareness that HIV can be transmitted through breastfeeding. Because the composite outcome could only be constructed for women with complete information on all three items, women with missing or incomplete responses to any of these questions were excluded from the analysis. After applying these exclusion criteria, the final analytical sample consisted of 5725 women.

### Study variables

The outcome variable was comprehensive knowledge of MTCT of HIV. This composite indicator was derived from three DHS questions assessing whether respondents knew that HIV can be transmitted from an infected mother to her child during (1) pregnancy, (2) delivery, and (3) breastfeeding. Each item had a binary response option (“Yes” or “No”).

A composite variable was created by combining these three knowledge items. Respondents who answered “Yes” to all three questions were classified as having good comprehensive knowledge of MTCT of HIV, whereas those who answered “No” to at least one of the three questions were classified as having poor comprehensive knowledge. Thus, the composite indicator was based on three variables representing knowledge of HIV transmission during pregnancy, delivery, and breastfeeding. This approach to defining the outcome variable has been widely used in previous DHS-based studies [12, 24–26]. Based on previous DHS-based studies on comprehensive knowledge of MTCT of HIV [12, 24–27], the following covariates were included: age (categorized as 15–19, 20–24, 25–29, 30–34, 35–39, 40–44, 45–49), region (clustered into seven groups: Cluster 1: Banadir; Cluster 2: Awdal, Woqooyi Galbeed, Togdheer, Sool, Sanaag; Cluster 3: Bari, Nugaal; Cluster 4: Galgaduud, Mudug; Cluster 5: Hiraan, Middle Shabelle; Cluster 6: Bay, Bakool; Cluster 7: Gedo, Lower Juba), residence (urban vs. rural/nomadic), education level (no education vs. primary and higher), frequency of listening to radio (at least once a week, less than once a week, not at all), frequency of watching television (at least once a week, less than once a week, not at all), and wealth index (poor, middle, rich).

In this study, we used R software version 4.0.2 for data management and analysis. Descriptive statistics were generated to summarize the sociodemographic variables and to present the prevalence of comprehensive HIV knowledge of MTCT among currently married women of reproductive age (15–49 years) in Somalia (Tables 1 and 2). To examine factors associated with comprehensive knowledge of MTCT of HIV, we employed multivariable logistic regression analysis. Logistic regression belongs to the class of limited dependent variable models and is appropriate when the dependent variable is binary. Since comprehensive knowledge of MTCT was categorized as good knowledge versus poor knowledge, a multivariable logistic regression model was fitted to identify factors independently associated with comprehensive MTCT knowledge while controlling for potential confounding variables [22, 23, 28]. Factors with a p-value less than 5% were considered statistically significant, while those with a p-value less than 10% were considered marginally significant. Adjusted odds ratios (AORs) with 95% confidence intervals (CIs) were used to assess the strength and direction of the associations.

**Table 1:** Demographic characteristics of respondents

Variables	levels	Frequency	Percentage
Age	15–19	438	7.65
	20–24	1014	17.71
	25–29	1366	23.86
	30–34	1129	19.72
	35–39	976	17.05
	40–44	520	9.08
	45–49	282	4.93
Regions	Cluster 1	844	14.74
	Cluster 2	2487	43.44
	Cluster 3	484	8.45
	Cluster 4	585	10.22
	Cluster 5	570	9.96
	Cluster 6	255	4.45
	Cluster 7	500	8.73
Residence	Rural and nomadic	2798	48.87
	Urban	2927	51.13
Education	No education	4418	77.17
	Primary and higher	1307	22.83
Frequency of listening to radio	At least once a week	489	8.54
	Less than once a week	215	3.76
	Not at all	5021	87.70
Frequency of watching Tv	At least once a week	722	12.61
	Less than once a week	215	3.76
	Not at all	4788	83.63
Wealth	Poor	1948	34.03
	Middle	2332	40.73
	Rich	1445	25.24

Cluster 1 (Banadir), Cluster 2 (Awdal, Woqooyi Galbeed, Togdheer, Sool, and Sanaag), Cluster 3 (Bari and Nugaal), Cluster 4 (Galgaduud and Mudug), Cluster 5 (Hiraan and Middle Shabelle), Cluster 6 (Bay and Bakool), and Cluster 7 (Gedo and Lower Juba)

**Table 2:** Prevalence of comprehensive and individual knowledge of MTCT of HIV of respondents

Variables	levels	Frequency	percentage
Can HIV be transmitted from Mother to her baby during pregnancy?	Yes	4357	76.10
	No	1368	23.90
Can HIV be transmitted from Mother to her baby during delivery?	Yes	4592	80.20
	No	1133	19.80
Can HIV be transmitted from Mother to her baby by breastfeeding?	Yes	4578	80.0
	No	1147	20.0
Comprehensive knowledge of MTCT of HIV	Poor	1908	33.33
	Good	3817	66.67

## Results

### Demographic characteristics

Table 1 summarizes the demographic characteristics of respondents in the study. The largest age group among respondents was 25–29 years (23.86%), followed by those aged 30–34 years (19.72%). The smallest age group was 45–49 years, accounting for only 4.93% of respondents. Regionally, Cluster 2 (Awdal, Woqooyi Galbeed, Togdheer, Sool, and Sanaag) was the most represented, with 43.44% of participants, while Cluster 6 (Bay and Bakool) had the smallest representation at 4.45%. Respondents were nearly evenly split between rural/nomadic (48.87%) and urban (51.13%) areas. Regarding education, a significant majority (77.17%) had no formal education, and only 22.83% attained primary or higher education. Media exposure was limited, with 87.70% of respondents never listening to the radio and 83.63% never watching television, highlighting the low penetration of media in the study population. Only a small fraction of participants listened to the radio (8.54%) or watched TV (12.61%) at least once a week. Wealth distribution revealed that 40.73% of respondents belonged to middle-income households, followed by 34.03% from poor households, and 25.24% from rich households.

### Prevalence of comprehensive and individual knowledge of MTCT of HIV

Table 2 presents the prevalence of individual and comprehensive knowledge of MTCT of HIV among currently married women of reproductive age (15–49 years) in Somalia. Overall, 76.1% of women reported that HIV can be transmitted from a mother to her baby during pregnancy, 80.2% recognized transmission during delivery, and 80.0% identified breastfeeding as a route of transmission. Based on these three knowledge components, a composite indicator of comprehensive MTCT knowledge was constructed. The results showed that 66.67% of women had good comprehensive knowledge of MTCT of HIV, indicating that they correctly identified all three modes of transmission, whereas 33.33% had poor comprehensive knowledge, meaning that they failed to identify at least one mode of transmission. These findings indicate that while knowledge of the individual routes of MTCT was relatively high, approximately one-third of currently married women in Somalia lacked comprehensive knowledge of all three modes of mother-to-child transmission of HIV.

### Factors associated with comprehensive knowledge of MTCT of HIV

Multivariable binary logistic regression was used to examine factors associated with comprehensive knowledge of MTCT of HIV among currently married women aged 15–49 years (Table 3). The results indicate that comprehensive knowledge was associated with demographic, geographic, socioeconomic, and media exposure factors. Region, wealth status, and frequency of listening to the radio were statistically significant at the 5% level, while age and frequency of watching television were significant at the 10% level. In terms of age, women aged 30–34 had higher odds of comprehensive MTCT knowledge compared to those aged 15–19; however, this association was not statistically significant at the 5% level (AOR = 1.22, 95% CI 0.97–1.55). Regarding regional differences, women in Cluster 7 had 61% lower odds of comprehensive MTCT knowledge (AOR = 0.39, 95% CI 0.31–0.50), while those in Cluster 6 had 47% lower odds (AOR = 0.53, 95% CI 0.39–0.71), both relative to women in Cluster 1.

**Table 3:** Multivariable logistic regression analysis of variables

Variables	Levels	Estimate	S. E	*P*-value	95% CI AOR		
					AOR	Lower bound	Upper bound
(Intercept)		1.16	0.19	0.00	3.21	2.22	4.65
Age	15–19(RC)	–	–	–	–	–	–
	20–24	0.14	0.12	0.25	1.15	0.91	1.45
	25–29	0.14	0.12	0.22	1.15	0.92	1.45
	30–34	0.20	0.12	0.09	1.22	0.97	1.55
	35–39	0.16	0.12	0.18	1.18	0.93	1.50
	40–44	0.08	0.14	0.58	1.08	0.82	1.42
	45–49	0.26	0.17	0.12	1.30	0.94	1.80
Regions	Cluster1(RC)	–	–	–	–	–	–
	Cluster2	− 0.26	0.10	0.01	0.77	0.64	0.94
	Cluster3	− 0.38	0.13	0.00	0.69	0.53	0.89
	Cluster4	− 0.52	0.12	0.00	0.59	0.47	0.75
	Cluster5	− 0.14	0.13	0.28	0.87	0.68	1.12
	Cluster6	− 0.64	0.15	0.00	0.53	0.39	0.71
	Cluster7	− 0.94	0.12	0.00	0.39	0.31	0.50
Residence	Urban (RC)	–	–	–	–	–	–
	Rural and nomadic	− 0.06	0.08	0.42	0.94	0.81	1.09
Education	No education (RC)	–	–	–	–	–	–
	Primary and higher	0.06	0.07	0.41	1.06	0.92	1.23
Frequency of listening to radio	At least once a week (RC)	–	–	–	–	–	–
	Less than once a week	− 0.15	0.19	0.41	0.86	0.60	1.24
	Not at all	− 0.34	0.11	0.00	0.71	0.57	0.88
Frequency of watching TV	At least once a week (RC)	–	–	–	–	–	–
	Less than once a week	0.01	0.17	0.96	1.01	0.73	1.41
	Not at all	0.17	0.10	0.09	1.18	0.97	1.44
Wealth	Poor (RC)	–	–	–	–	–	–
	Middle	− 0.04	0.08	0.61	0.96	0.82	1.12
	Richest	− 0.33	0.10	0.00	0.72	0.59	0.87

*S.E* Standard Error, *AOR* Adjusted Odds Ratio, *CI* Confidence Interval, *RC* Reference category

Media exposure was also significantly associated with MTCT knowledge. Women who did not listen to the radio had 29% lower odds of comprehensive knowledge compared to those who listened at least once a week (AOR = 0.71, 95% CI 0.57–0.88). Finally, wealth status showed an inverse relationship, where women in the richest quintile had 28% lower odds of comprehensive knowledge compared to those in the poorest quintile (AOR = 0.72, 95% CI 0.59–0.87).

## Discussion

The purpose of this study was to assess comprehensive knowledge of MTCT of HIV and its associated factors among currently married women aged 15–49 years in Somalia. The analysis identified key determinants of HIV knowledge, including access to media (particularly radio and television), wealth status, age, and region of residence. These findings are consistent with results from similar studies conducted in other SSA countries. In this study, 66.67% of currently married women demonstrated comprehensive knowledge of MTCT. However, this proportion is lower than those reported in previous studies from other SSA countries, including Zimbabwe [29] and Nigeria [30], highlighting potential gaps in awareness and access to HIV-related information in the Somali context.

The study revealed that older women were more likely to possess comprehensive HIV knowledge about MTCT and its prevention than their younger counterparts aged 15–19 years, with women aged 30–34 years showing marginally higher odds of comprehensive knowledge at the 10% significance level. This finding is consistent with previous research conducted in sub-Saharan Africa, including Ethiopia and Zimbabwe [26, 29, 31]. One possible explanation is that older women tend to have more frequent interactions with maternal health services due to repeated pregnancies, which increases their exposure to HIV-related information. Furthermore, the study highlights that current HIV education and prevention strategies may not sufficiently address the needs of younger women, particularly adolescents. A lack of supportive environments and limited access to accurate information may hinder young women’s ability to engage in essential preventive behaviors, such as adherence to ART. The study also found that women in the richest wealth category had lower odds of possessing comprehensive MTCT knowledge of HIV compared to those in the poorest category. This finding contrasts with the majority of existing research in SSA [9, 10, 12, 24, 29, 32, 33], where a positive association between wealth and HIV knowledge is typically observed. One possible explanation for the inverse association observed in this study is that HIV education and awareness programs in some settings are primarily delivered through community-based and public health interventions that often target socioeconomically disadvantaged populations [14, 34, 35]. Consequently, women from wealthier households may have fewer opportunities to participate in such programs and may receive less exposure to HIV-related educational messages. Previous studies have also suggested that access to HIV information is influenced by multiple social and contextual factors beyond economic status alone populations [14, 34, 35]. Therefore, greater household wealth may not necessarily translate into higher levels of comprehensive knowledge of MTCT of HIV.

The study further found that women in Clusters 6 (Bay and Bakool) and 7 (Gedo and Lower Juba) had substantially lower odds of MTCT knowledge (47–61% lower). These regions are affected by ongoing insecurity and limited humanitarian access [10, 11]. Health infrastructure in these areas is severely constrained, with fewer functional health facilities providing antenatal care and HIV testing services. As a result, women in these regions are less likely to receive routine maternal health education, counselling on HIV prevention, or exposure to structured health promotion messages. Limited outreach services and reduced mobility due to insecurity further restrict contact with health providers and community health programs, thereby reducing opportunities for health information dissemination. These findings are consistent with previous studies [17, 36–38] that have reported lower levels of MTCT knowledge of HIV in conflict-affected and hard-to-reach settings, where insecurity, weak health systems, and limited access to maternal and child health services limit exposure to essential health information. Lastly, our study found that women who do not listen to the radio have lower odds of possessing a higher level of comprehensive MTCT knowledge about HIV. This result aligns with research from Sub-Saharan Africa, which demonstrates that exposure to mass media, such as radio and television, is linked to better HIV knowledge [15, 39, 40]. These media platforms are essential tools for disseminating crucial information to the public. While previous studies have targeted different populations, such as youth and males, our findings are consistent, underscoring the significant role of mass media in enhancing public awareness and education about HIV, particularly in relation to vertical transmission of HIV.

### Strengths and limitations

This study has strengths and limitations. A key strength is its contribution to the existing evidence on knowledge of MTCT of HIV among married women in Somalia. By using nationally representative data from the 2018–2019 SDHS, the study provides robust and generalizable insights into HIV-related knowledge in this population. However, several limitations should be considered. First, the cross-sectional design limits the ability to establish causal relationships, as MTCT knowledge was assessed at a single point in time. Second, the use of self-reported data may have introduced recall and social desirability biases, potentially affecting the accuracy of responses. Third, due to security-related exclusions of some regions, the findings may not fully represent the entire Somali population. Fourth, the analysis was restricted to variables available in the SDHS dataset. Potentially relevant factors, including pregnancy history, maternal healthcare utilization, and cultural and political influences, could not be assessed because of data limitations. Future research should consider these determinants to provide a more comprehensive understanding of factors associated with MTCT knowledge. Fifth, the outcome variable was constructed as a composite measure of comprehensive MTCT knowledge based on three questions assessing HIV transmission during pregnancy, delivery, and breastfeeding. These items were used because they are the only MTCT-related indicators available in the SDHS dataset and represent the standard DHS approach for measuring knowledge of mother-to-child transmission.

## Conclusion

The findings of the study indicate that comprehensive knowledge of MTCT of HIV among currently married women in Somalia is associated with age, wealth status, geographic region, and media exposure. These findings may inform policy efforts aimed at strengthening MTCT of HIV awareness through targeted health education interventions. Particular attention could be directed toward younger women, those with limited access to radio and television, and women residing in Bay, Bakool, Gedo, and Lower Juba. Expanding community-based and facility-based HIV education, potentially through antenatal care services, may be beneficial. Additionally, addressing socioeconomic and regional disparities by improving access to health information for low-income groups and underserved areas could contribute to more equitable dissemination of HIV prevention knowledge. Future research is needed to further explore the determinants of MTCT knowledge and to evaluate the effectiveness of targeted interventions in the Somali context.

## Data Availability

The data used in this study are derived from the SHDS 2018–19, conducted by the Somali National Bureau of Statistics . The dataset is publicly available upon request from the Somali National Bureau of Statistics through the official microdata portal: https://microdata.nbs.gov.so/index.php/catalog/50. Researchers can access the data after registration and approval from the data provider.
